# An association of CSF apolipoprotein E glycosylation and amyloid-beta 42 in individuals who carry the APOE4 allele

**DOI:** 10.1186/s13195-023-01239-0

**Published:** 2023-05-23

**Authors:** Cristiana J. Meuret, Yueming Hu, Sabrina Smadi, Mikaila Ann Bantugan, Haotian Xian, Ashley E. Martinez, Ronald M. Krauss, Qiu-Lan Ma, Dobrin Nedelkov, Hussein N. Yassine

**Affiliations:** 1 University of Southern California, 2250 Alcazar St, Rm 210, Los Angeles, CA 90033 USA; 2 Isoformix Inc., 9830 S. 51. St. Suite B-113, Phoenix, AZ 85044 USA; 3 University of California, San Francisco, CA USA

**Keywords:** Apolipoprotein E, Isoform, Glycan, Mass spectrometry, Alzheimer’s disease

## Abstract

Carrying the apolipoprotein E (ApoE) Ɛ4 allele is associated with an increased risk of cerebral amyloidosis and late-onset Alzheimer’s disease, but the degree to which apoE glycosylation affects its development is not clear. In a previous pilot study, we identified distinct total and secondary isoform-specific cerebral spinal fluid (CSF) apoE glycosylation profiles, with the E4 isoform having the lowest glycosylation percentage (E2 > E3 > E4). In this work, we extend the analysis to a larger cohort of individuals (*n* = 106), utilizing matched plasma and CSF samples with clinical measures of AD biomarkers. The results confirm the isoform-specific glycosylation of apoE in CSF, resulting from secondary CSF apoE glycosylation patterns. CSF apoE glycosylation percentages positively correlated with CSF Aβ_42_ levels (*r* = 0.53, *p* < 0.0001). These correlations were not observed for plasma apoE glycosylation. CSF total and secondary apoE glycosylation percentages also correlated with the concentration of CSF small high-density lipoprotein particles (s-HDL-P), which we have previously shown to be correlated with CSF Aβ_42_ levels and measures of cognitive function. Desialylation of apoE purified from CSF showed reduced Aβ_42_ degradation in microglia with E4 > E3 and increased binding affinity to heparin. These results indicate that apoE glycosylation has a new and important role in influencing brain Aβ metabolism and can be a potential target of treatment.

**Supplementary Information:**

The online version contains supplementary material available at 10.1186/s13195-023-01239-0.

## Introduction

The three major alleles of the *APOE* gene (Ɛ2, Ɛ3, Ɛ4) give rise to three apoE isoforms (E2, E3, and E4), which differ in their ability to bind to lipids, receptors, and amyloid-β (Aβ) [1–7]. The Ɛ4 allele significantly increases the risk for both cerebral amyloidosis and late-onset Alzheimer’s disease (AD), but the mechanistic basis for the contribution of the E4 isoform to the development of AD remains unclear. In the brain, apoE acquires lipids to form high-density lipoprotein (HDL)-like particles that vary in size and functional properties. Among the various sizes, small HDL (s-HDL) sized between 7 and 10 nm have cholesterol efflux functional properties, associate with less cerebral amyloidosis, and correlate positively with performance on cognitive domains [8].

Some recent findings suggest a role for apoE glycosylation in Aβ plaque formation [9–11]. ApoE is glycosylated with an *O*-linked *N*-acetylgalactosamine-galactose disaccharide (-GalNAc-Gal), onto which one or two sialic acids can be further added enzymatically in vivo [12]. The two most common glycosylation sites of apoE are Thr^194^ [13] and Ser^290^ [14], although additional possible glycan attachments sites have also been identified, including Thr^8^, Thr^18^, Ser^197^, Ser^263^, Thr^289^, and Ser^296^ [9, 15–17]. Chua et al. found that distinctive patterns of apoE glycosylation correlated with an increase of Aβ_42_ in the brains of mice with Niemann-Pick type C (NPC) disease before any neurological abnormalities were found. Using lectin to bind complex oligosaccharides, they discovered that apoE glycosylation patterns have distinctive changes with disease progression; however, the specifics of these pattern changes could not be determined [10]. In a more recent study with a small cohort of healthy individuals, Flowers et al. determined that plasma apoE lacked glycosylation at the C-terminus, whereas the matched CSF samples revealed apoE’s C-terminus to be extensively glycosylated and sialylated [9]. This corroborated previous findings that CSF apoE is more glycosylated than plasma apoE [18] and that the removal of sialic acids (desialylation) from apoE reduces its binding affinity for HDL [19]. Sialyltransferases, the enzymes that couple sialic acid to the core apoE’s *O*-glycan, were found to decrease with both age [20] and AD [21], possibly affecting apoE’s ability to bind to HDL.

We developed an accurate mass spectrometric immunoassay (MSIA) for apoE isoform and glycoform detection, applied it to plasma and CSF samples of a cognitively normal cohort (*n* = 22), and identified isoform-specific glycosylation patterns in matched plasma and CSF [22]. In the present study, we extend these observations to a larger cohort of individuals that differ by cognitive status and with measurements of AD biomarkers. We report strong associations between the degree of apoE glycosylation in CSF and CSF Aβ_42_ independent of cognitive status. We hypothesize that the extent to which CSF apoE is sialylated affects receptor binding and the formation of s-HDL-P with downstream implications for the development of AD pathology before the onset of clinical symptoms.

## Materials and methods

### Human samples

EDTA plasma and CSF samples from a cohort of 106 individuals were analyzed (Table 1; included in this cohort were the 22 individuals from our first study [22]). Recruitment methods were directed at persons enrolled in the University of Southern California (USC) Alzheimer Disease Research Center (ADRC) enrolled continuously between 2010 and 2022 and targeting cognitively normal individuals at greater risk of AD. All participants completed the NACC Uniform Data Set that includes a standard battery of neuropsychological testing and dementia risk factors. To assess cognitive status, we utilized both the clinical dementia rating (CDR) test and the Neuropsychological Evaluation from Uniform Data Set (Versions 2 or 3) of the National Alzheimer’s Coordinating Center. The Neuropsychological Evaluation covered multiple cognitive domain considerations. The diagnosis of probable dementia was made using a consensus conference. Included were participants ≥ 40 years of age with neuropsychologically confirmed absence of cognitive dysfunction and/or cognitive dysfunction, with no current or prior history of any neurological or psychiatric conditions that might contribute to any observed cognitive impairment, including organ failure, brain tumors, epilepsy, hydrocephalus, schizophrenia, and major depression. The study and procedures were approved by the Institutional Review Board of USC. All participants provided informed consent prior to enrollment in the study (USC IRB: HS-16–00,888). Samples were obtained fasting and processed within 2 h after collection and were immediately frozen at – 80 °C.

**Table 1:** Study cohort’s demographic and clinical characteristics

Characteristic	*N*	*N* = 106
Age, mean (range)	105	70.1 (47.0, 91.0)
Unknown		1
Gender, *n*/*N* (%)	106	
Female		61/106 (58%)
Male		45/106 (42%)
Race, *n*/*N* (%)	105	
African American		5/105 (4.8%)
Asian or Pacific Islander		11/105 (10%)
Caucasian		83/105 (79%)
Native American		4/105 (3.8%)
Other		2/105 (1.9%)
Unknown		1
Education (years), mean (range)	79	16.05 (10.00, 21.00)
Unknown		27
CDR score, *n*/*N* (%)	94	
0		57/94 (61%)
0.5		31/94 (33%)
1		2/94 (2.1%)
2		2/94 (2.1%)
3		2/94 (2.1%)
Unknown		12
APOE genotype, *n*/*N* (%)	106	
22		1/106 (0.9%)
23		9/106 (8.5%)
24		5/106 (4.7%)
33		52/106 (49%)
34		25/106 (24%)
44		14/106 (13%)
Clinical status, *n*/*N* (%)	106	
NCI		74/106 (70%)
MCI		16/106 (15%)
AD		16/106 (15%)
Homozygous Ɛ4 clinical status, *n*/*N* (%)	14	
NCI		11/14 (79%)
MCI		1/14 (7.1%)
AD		2/14 (14%)
CSF AB42 (pg/mL), median (IQR)	90	280 (163, 599)
Unknown		16
CSF total tau (pg/mL), median (IQR)	88	318 (213, 455)
Unknown		18
pTau (pg/mL), median (IQR)	83	59.7 (44.8, 78.8)
Unknown		23

### ApoE mass spectrometric immunoassay (MSIA)

The apoE assay was performed as described previously [22]. MSIA Tips derivatized with apoE antibody were used to affinity retrieve apoE from either plasma (40 μL) or CSF (200 μL). The polyclonal goat anti-human antibody to apoE (Cat. No. 50A-G1) was obtained from Academy Biomedical (Houston, TX); the antibody does not have isoform bias, as evidenced from the results in which the isoforms ratios are similar to those obtained with antibody-free approaches. ApoE eluted from the MSIA Tips was analyzed with a MALDI-TOF mass spectrometer (Autoflex III MALDI-TOF, Bruker, Billerica, MA), operated in positive ion mode, with a mass spectra range from 7 to 70 kDa, 700 ns delay, 20.00 kV and 18.45 kV ion source voltages, and signal suppression of up to 7000 Da. The spectra were baseline subtracted (Convex Hull algorithm, 0.8 flatness) and smoothed (Savizky Golay algorithm, 5 m/z width, and 1 cycle) using Flex Analysis software (Bruker Daltonics). The peak intensities of all isoforms and glycoforms were measured using Zebra 1.0 software (Intrinsic Bioprobes Inc.) and tabulated in a spreadsheet. For samples from heterozygous individuals, peak intensities were measured separately for the two isoforms and their corresponding glycoforms in each mass spectrum. The intensities of all apoE peaks (unglycosylated protein and all glycoforms) were separately summed for each isoform, and the peak intensity of each apoE isoform-specific signal was divided by the summed intensity of all peaks for that isoform, to obtain the percent abundance. The glycoforms percent abundance for a specific isoform was summed and then divided by the total percent abundance for that isoform to obtain the total glycosylation percentage. Similarly, percent abundances of the secondary glycosylation glycoforms were summed and divided by the total percent abundance for that isoform.

### CSF AD biomarkers

CSF levels of Aβ_42_, tau, and phosphorylated tau (pTau) were measured using Meso Scale Discovery (MSD) [23] multiplex assay.

### HDL particle measurements using ion mobility (IM)

Concentrations of HDL-P were measured by ion mobility (IM) after treatment with dextran sulfate to remove non-lipid bound proteins such as albumin from 30 μL of plasma or CSF, as described previously [8]. For the analyses described here, particles in the HDL size range were classified as small (7.0–10.5 nm) and total (7–14.5 nm) HDL. The coefficient of variation for HDL-P measured by IM was less than 16% [24].

### Preparation of desialylated and sialylated samples

A total of 14 mL of CSF was pooled from homozygous Ɛ3 or Ɛ4 participants, 7 mL of which were incubated with 1 mL of sialidase (Sigma, Cat. No. 11585886001) (1 unit) on an incubator (250 rpm, 2 h, 37 °C). The sialidase-treated aliquot and the remaining untreated aliquot were then separately incubated with a polyclonal goat anti-human apoE antibody (Academy Biomedical, Cat. No. 50A-G1) coupled to 100 μL of agarose resin (Pierce™ NHS-Activated Slurry, Thermo Fisher, Cat. No. 26200) overnight at 4 °C on a rotator. The samples were then equilibrated to room temperature and transferred to Bio-spin Disposable Chromatography Columns (BIO-RAD, Cat. No. 732–6008). The columns were first washed with 10 bed volumes of 1 $\times$ PBS, and then eluted with 10 bed volumes of 0.5% trifluoroacetic acid (TFA) into neutralizing buffer (50 mM ammonium acetate, pH = 9.25). The samples were partially evaporated via rotary evaporation, and then diluted in a 1:1 ratio with 40 mM Tris–HCl for a final concentration of 20 mM Tris–HCl (pH = 7.4) in preparation for the heparin binding experiments. Due to the requirements of large volumes of CSF to isolate apoE, only 1 batch of pooled CSF was isolated for the in vitro experiments from a cognitively normal Ɛ3 or Ɛ4 homozygote. The effectiveness of isolated apoE desialylation was assessed with MSIA as described below.

### Evaluation of desialylated and sialylated apoE function in vitro

To understand the biological function of desialylated and sialylated apoE, we investigated the impact of removing apoE sialylation on oligomeric Aβ_42_ in cultured immortalized BV2 murine microglial cells. The oligomeric Aβ_42_ was prepared as previously described with a mild modification [25]. Briefly, the lyophilized HiLyte™ Fluor 488-labeled human Aβ_42_ peptide (AnaSpec Inc., Cat.AS-60479–01) was first dissolved in DMSO at a concentration of 3 mg/mL and aliquoted to store at − 80 °C freezer until use. During the experiment period, the Aβ_42_ was diluted with 1 $\times$ PBS (pH = 7.4) to a final concentration of 221 μM. This solution was incubated at room temperature with a micro stir bar at 550 rpm for 16 h. For the cell culture experiment, the BV2 cells were seeded in 96-well plates at a density of 0.2 $\times$ 10^5^ in Dulbecco’s modified Eagle’s medium (DMEM, Corning, 17–205-CV) supplemented with 10% fetal bovine serum (Omega Scientific, FB-12) and 1% of antibiotic–antimycotic (Anti-anti, Thermo Fisher, Catalog number 15240062) in a 5% CO_2_-humidified air environment incubator at 37 °C. On the next day, the culture media was removed and replaced with a serum free DMEM at the time of treatment with tested agents. The cells were treated with and/or without 0.2 µM of Fluor 488-labeled oligomeric Aβ_42_, 10 nM of sialylated and desialylated apoE3 or apoE4 for 1.15 h. Then, Aβ_42_ fluorescent intensity was measured by SpectraMad®iD5 spectrophotometer.

### HiTrap heparin chromatography

A 1 mL HiTrap heparin column (Cytiva, Cat. No. 17040601) was washed with 10 mL of MQ H_2_O followed by an equilibration step with 10 mL of 20 mM Tris–HCl buffer at physiological pH (1 mL/min). The performance of the column was first optimized with recombinant apoE3. Then, a 1.96 mL of desialylated E3 CSF sample was applied to the column and recycled through it 5 times manually at 1 mL/min using a syringe pump (Pump 11 Elite, Harvard Apparatus). The heparin column was then incubated for 10 min before connecting it to the Bio-Rad NGC Chromatography system. The column was first washed with 10 bed volumes of the 20 mM Tris–HCl buffer, and then eluted at 1 mL/min with a NaCl gradient in 20 mM tris–HCl (pH = 7.4) [0.5–1 M, 2.5% increase per fraction, 1 mL per fraction]. The column was next washed with 10 mL of 2 M NaCl, followed by 10 mL of 20 mM tris–HCl and then stored in 20% ethanol. Fractions were collected and analyzed using an in-house apoE enzyme-linked immunoassay (ELISA). The same method was used for the sialylated E3 CSF sample.

### Data analysis

Statistical analysis was performed using RStudio (http://www.R-project.org/). Unless otherwise indicated, an alpha level of 0.05 was used to determine statistical significance. For normally distributed data, as determined by non-significant *p*-values from the Shapiro–Wilk test, a one-way ANOVA model was used to test overall group differences, followed by a post hoc Tukey HSD test for between-group comparisons to identify differences among isoforms and between Ɛ4 vs non- Ɛ4 participants. Non-normally distributed data, as determined by significant *p*-values from the Shapiro–Wilk test, were analyzed using linear regression models. Model residuals were evaluated for normality and homoscedasticity. The method used to calculate correlation coefficients in all the scatterplots was Spearman. Finally, pairwise Wilcoxon rank sum tests were used to determine differences in non-normally distributed data for between-group comparison, and *p*-values were adjusted via the Bonferroni method for multiple comparisons.

## Results

Isoform-specific apoE glycosylation patterns in matched plasma and CSF were identified within a cohort of 106 individuals (Table 1). A total of 71% of the cohort had no evidence of cognitive impairment, 14% had mild cognitive impairment (MCI), and 15% had probable AD. Since our recruitment efforts were focused on targeting cognitively normal individuals at greater risk of AD, a disproportionately greater percentage of cognitively normal *APOE* Ɛ4 carriers participated. 6 In the entire cohort, 14 were Ɛ4 homozygotes, and out of those, 11 were cognitively normal.

**Fig. 1:**
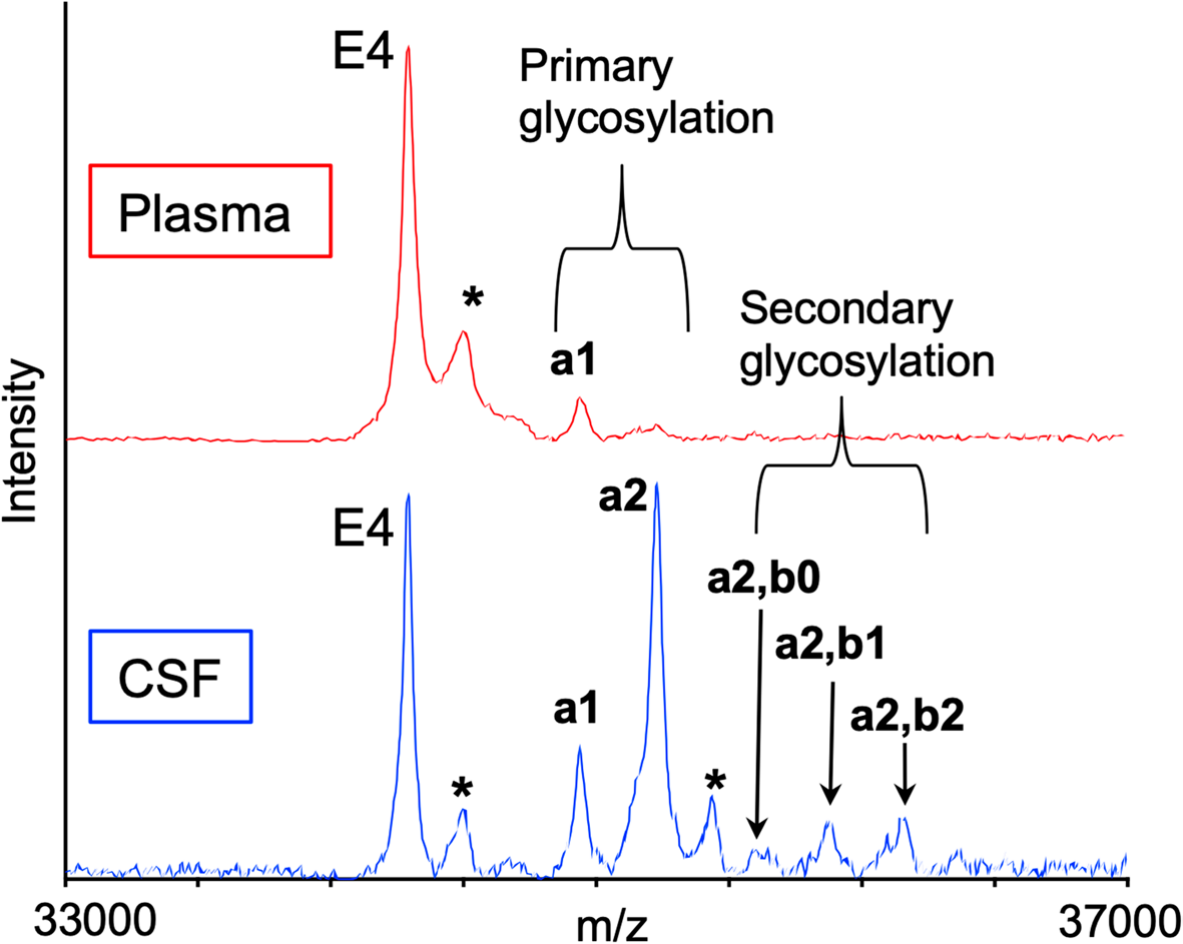
Representative mass spectra resulting from the analysis of matched plasma and CSF from homozygous Ɛ4/Ɛ4 individual. Matrix adduct peaks are labeled with *. Glycan peak labeling: a1—GalNAc-Gal-Sia; a2—GalNAc(-Sia)-Gal-Sia; a2, b0—GalNAc(-Sia)-Gal-Sia, GalNAc-Gal; a2, b1—GalNAc(-Sia)-Gal-Sia, GalNAc-Gal-Sia; a2, b2—GalNAc(-Sia)-Gal-Sia, GalNAc(-Sia)-Gal-Sia

A single *O*-linked glycan attached to apoE (most likely at the primary glycosylation site, Thr^194^) [13], was observed in all mass spectra obtained from plasma samples, while two glycans per apoE were observed in mass spectra obtained from CSF (Fig. 1), indicating a secondary glycosylation, which is an additional glycosylation of an already singly-glycosylated apoE, at a site that is different from the primary glycosylation site (most likely on the C-terminus). Hence, in CSF both total glycosylation percentage (from both sites), and secondary glycosylation alone were analyzed and presented. The glycosylation percentages by clinical status (AD, MCI, and NCI) are shown in Table 2; there were no statistically significant differences in plasma; however, CSF total glycosylation was higher in the MCI vs NCI group (*p* = 0.005) and CSF secondary glycosylation was higher in the MCI vs AD group (*p* = 0.007), as well as higher in the MCI vs NCI group (*p* < 0.001). The difference in CSF secondary glycosylation between AD and NCI group was not significant likely because the NCI group included 11 samples from individuals who were Ɛ4 homozygous. The glycosylation data as a function of clinical status in non- Ɛ4 vs Ɛ4 participants, along with the biomarkers data, are also presented in Fig. 1S.

**Table 2:** Distribution of apoE glycosylation percentages in both plasma and CSF and AD biomarkers by clinical status of the participants

	AD dementia (*n* = 16)	MCI (*n* = 16)	NCI (*n* = 74)	*p*-value
Plasma total glycosylation %	10.7 (9.10, 13.6)	11.6 (9.83, 14.2)	12.4 (10.3, 15.4)	0.98
CSF total glycosylation %	69.9 (3.69)	71.3 (3.56)	67.6 (3.95)	0.003^a^
CSF secondary glycosylation %	22.6 (4.69)	27.6 (4.08)	21.8 (3.83)	< 0.001^a,c^
CSF Aβ_42_ (pg/mL)	291 (195, 478)	800 (597, 933)	223 (149, 457)	< 0.001^a,c^
CSF Tau (pg/mL)	486 (333, 588)	215 (160, 284)	336 (232, 475)	0.002^a,c^
pTau (pg/mL)	84.8 (78.0, 106)	63.0 (46.1, 74.4)	54.3 (42.0, 73.3)	< 0.001^b,c^

Glycosylation/biomarker data are means (SD), for normally distributed data, and medians (IQR), for non-normally distributed data

*MCI*, mild cognitive impairment; *NCI*, no cognitive impairment

^a^NCI-MCI: CSF total glycosylation % (*p* = 0.005), CSF secondary glycosylation % (*p* < 0.001), CSF Aβ_42_ (*p* < 0.001), CSF tau (*p* = 0.006)

^b^NCI-AD: pTau (*p* = 0.004)

^c^MCI-AD: CSF secondary glycosylation (*p* = 0.007), CSF Aβ_42_ (*p* = 0.013), CSF tau (*p* = 0.011), pTau (*p* = 0.017)

**Fig. 2:**
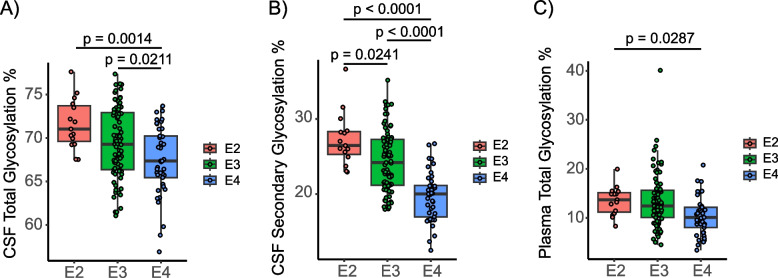
The apoE4 isoform exhibited lower percentage of CSF secondary glycosylation. Percent glycosylation by individual apoE isoforms in CSF (total and secondary) and plasma from the study cohort (*n* = 106). ApoE isoform-specific glycosylation was computed by dividing the peak intensity of the glycosylated forms with the total apoE peak intensity for each isoform. The groups were compared using a linear regression model

**Fig. 3:**
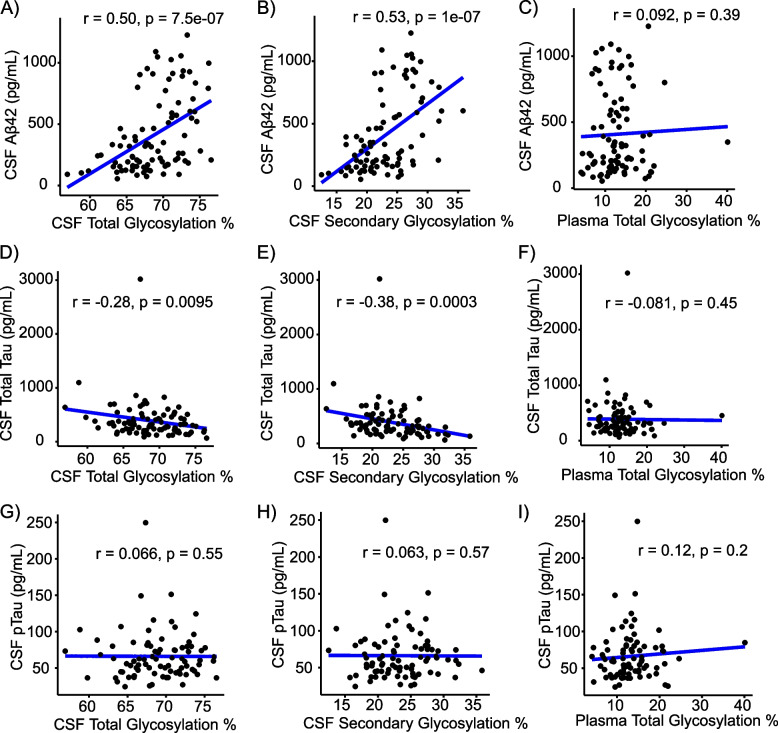
CSF secondary glycosylation correlates with CSF Aβ_42_ and CSF total tau levels. Correlations of CSF (total and secondary) and plasma glycosylation percentages with CSF Aβ_42_ (n=90), CSF pTau (n=83), and CSF total tau concentrations (*n* = 88). Spearman correlation coefficients were obtained

In both plasma and CSF, the percentage of total (Fig. 2A) and secondary (Fig. 2B) glycosylated apoE was lower in an isoform-dependent manner (E2 >  E3 >  E4). A small trend of lower glycosylation was observed in plasma (Fig. 2C). The mean (standard deviation) plasma glycosylation percentage was 13.5% (2.94%) glycosylated for apoE2, 13.4% (5.41%) glycosylated for apoE3, 10.3% (3.83%) glycosylated for apoE4, with statistically significant differences between E4 vs. E2 (*p* = 0.0287). When total CSF glycosylation was considered, mean (SD) glycosylation percentage was 71.6% (2.9%) glycosylated for apoE2, 69.4% (4.04%) glycosylated for apoE3, 67.4% (3.96%) for glycosylated apoE4, with statistically significant differences between glycosylated E4 vs. E2 (*p* = 0.0014) and E4 vs. E3 (*p* = 0.0211). When only the secondary CSF apoE glycosylation was considered, the mean (SD) was 27.3% (3.57%) glycosylated for apoE2, 24.4% (4.04%) glycosylated for apoE3, 19.6% (3.3%) glycosylated for apoE4, with statistically significant differences observed among all glycosylated apoE isoforms (E4 vs. E2, *p* < 0.0001; E4 vs. E3, *p* < 0.0001; E3 vs. E2, *p* = 0.0241). The glycosylation data plotted as a function of the genotype are shown in Fig. 2S. Furthermore, the overall glycosylation of apoE in CSF was much higher than in plasma (mean (SD)) with 68.5% (4.07%) in CSF vs. 13.0% (5.11%) in plasma (*p* < 0.0001) (Fig. 3S).

CSF Aβ_42_ levels (pg/mL) correlated with total (*r* = 0.50, *p* < 0.0001, Fig. 3A) and secondary (*r* = 0.53, *p* < 0.0001, Fig. 3B) CSF apoE glycosylation percentages, but not with plasma total glycosylation percentages (Fig. 3C). Similarly, CSF total tau (pg/mL) correlated with total (*r* =  − 0.28, *p* = 0.0095) (Fig. 3D) and secondary (*r* =  − 0.38, *p* = 0.0003) (Fig. 3E) CSF apoE glycosylation percentages, but not with plasma total glycosylation percentage (Fig. 3F). CSF pTau did not show any correlation with either CSF or plasma apoE glycosylation percentages (Fig. 3G–I).

**Fig. 4:**
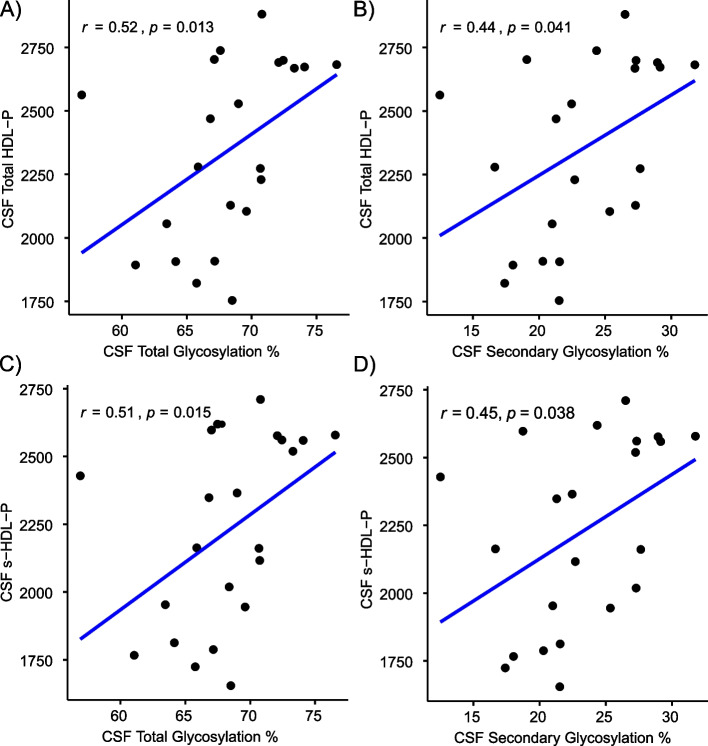
The percentage of CSF apoE secondary glycosylation correlates with CSF small HDL particle (s-HDL-P) concentrations. Correlations of CSF total and secondary glycosylation percentages with s-HDL-P and total CSF HDL-P were obtained in a subset of the cohort (n=22). Spearman correlation coefficients were obtained

CSF total (*r* = 0.52, *p* = 0.013, Fig. 4A) and secondary (*r* = 0.44, *p* = 0.041, Fig. 4B) glycosylation percentages correlated with total CSF HDL-P concentration (*n* = 22) (Fig. 4). Furthermore, CSF total (*r* = 0.51, *p* = 0.015, Fig. 4C) and secondary (*r* = 0.45, *p* = 0.038, Fig. 4D) glycosylation percentages correlated with s-HDL-P concentration, but not with large HDL-P (l-HDL-P) (data not shown). The association between s-HDL-P and CSF Aβ_42_ levels is shown in Fig. 4S.

**Fig. 5:**
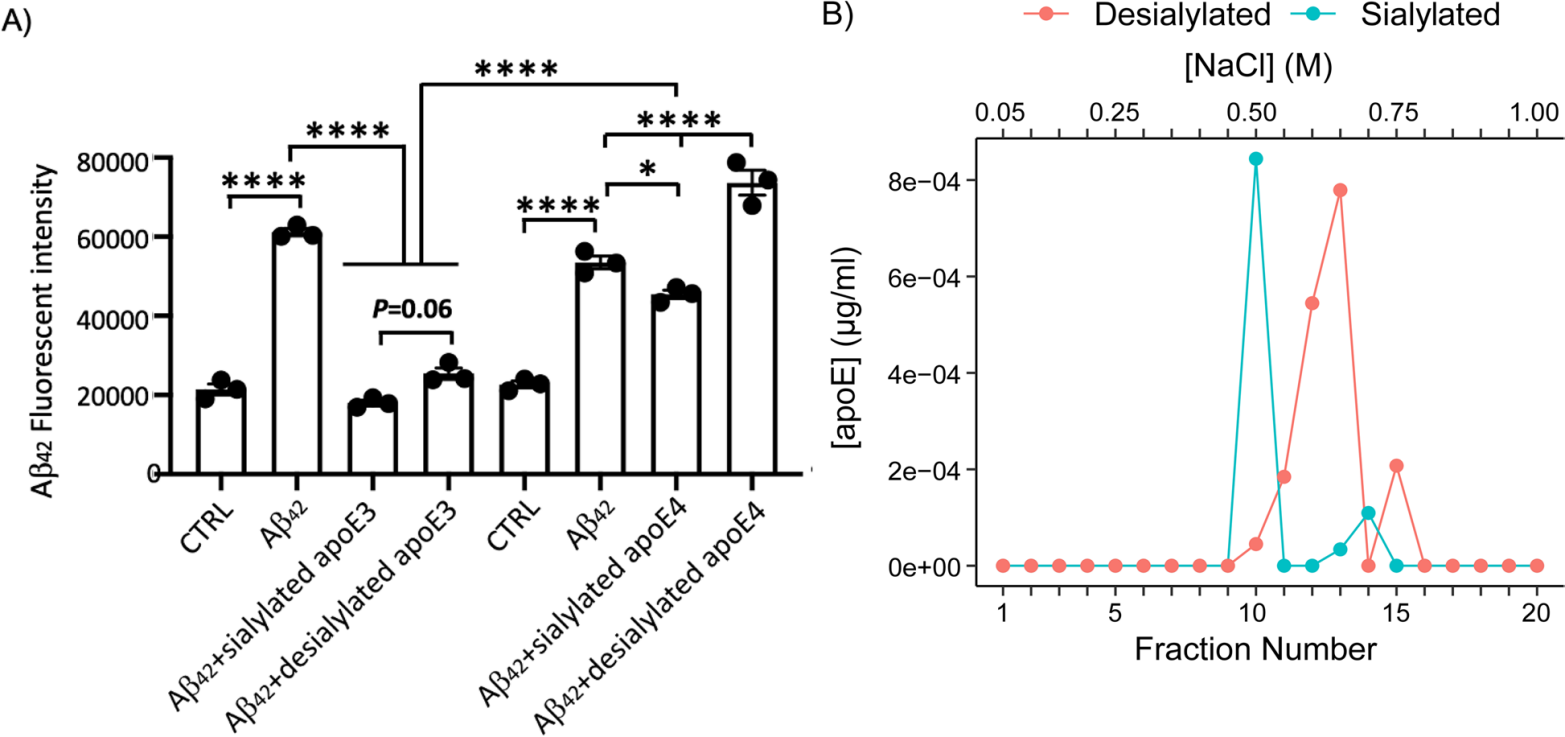
**A** Sialylated apoE mediated degradation of oligomeric Aβ_42_ in BV2 microglial cells. Aβ_42_ fluorescent intensity was measured by SpectraMad®iD5 spectrophotometer. In Aβ_42_-treated cells, Aβ_42_ fluorescent intensity was increased compared to the groups without Aβ_42_ (control/CTRL). Sialylated apoE3, apoE4, and desialylated apoE3 reduced the elevation of Aβ_42_ fluorescent intensity but desialylated apoE4 appeared to increase Aβ_42_ levels (*n* = 3). The data was quantified by one-way ANOVA. **B** ApoE glycosylation affects heparin binding. Elution of fully sialylated apoE3 isolated from CSF, and desialylated apoE3, from a heparin column from a pooled sample of several Ɛ3/Ɛ3 individuals. *p < 0.05, ****p < 0.0001

ApoE3 was isolated from Ɛ3/Ɛ3 CSF (one batch) and desialylated using sialidase (Fig. 5S). To understand the biological functional difference between desialylated and sialylated apoE, we investigated the impact of desialylated (10 nM) and sialylated apoE3 and apoE4 (10 nM) on oligomeric Aβ_42_ (0.2 µM) levels in cultured BV2 murine microglial cells. Compared to oligomeric Aβ_42_-treated cells, both sialylated and desialylated apoE3 significantly reduced Aβ_42_ fluorescent intensity in microglial cells (*p* < 0.0001) with sialylated apoE3 having a greater effect than desialylated apoE3 (*p* = 0.06). Although we observed a reduction of Aβ_42_ fluorescent intensity by sialylated apoE4 in microglial cells (*p* < 0.05), it had much less effect than sialyated apoE3 (*p* < 0.0001). Unlike desialylated apoE3, desialylated apoE4 significantly increased Aβ_42_ fluorescent intensity compared to desialylated apoE3, sialylated apoE4, or oligomeric Aβ_42_-treated cells (*p* < 0.0001, Fig. 5A). This data suggests that sialylation of apoE3 and apoE4 might influence the degradation of oligomeric Aβ_42_ whereas desialylation of apoE4 might influence the accumulation or aggregation of Aβ_42_. Following optimization of the heparin column using recombinant apoE3, fully sialylated and desialylated apoE3 isolated from CSF were analyzed and their elution times from the column compared. Sialylated apoE3 eluted with 0.5 M NaCl, while desialylated apoE3 eluted with 0.65 M NaCl (Fig. 5B). These results demonstrate that apoE desialylation increases heparin binding.

## Discussion

In this work, we confirmed isoform-specific apoE glycosylation profiles, with a lower percentage of apoE4 glycosylation in CSF, but not in plasma (Fig. 2). The primary glycosylation site of apoE is at Thr^194^ [13], while the secondary glycosylation site is on the C-terminus [14]. Within apoE4 (Arg^112^, Arg^158^), a salt bridge exists between Arg^112^ and Glu^109^ that leads to domain interactions between Arg^61^, on the N-terminus, and Glu^255^, on the C-terminus, giving it a more closed “hairpin” morphology compared to apoE2 and apoE3 [26]. However, this Ɛ4-isoform distinct morphology alone does not explain the apoE isoform-specific glycosylation profiles, because those profiles were observed only in CSF and not in plasma. Structurally, the isoforms have distinct three-dimensional conformations, and these differences may influence the probability of glycan binding to apoE glycosylation residues in CSF. It is also possible that the isoform-specific CSF glycosylation profiles are the result of reduced sialyltransferase activity in the brain. A decrease in sialyltransferase activity has been suggested as a biomarker for AD [21, 27]. Furthermore, global protein glycosylation in AD brains has been shown to decrease in the frontal lobe and increase in the hippocampus, while total protein glycosylation in serum was reduced [28]. To date, no comparable analysis of glycosylation and sialylation enzyme activity has been performed among the apoE isoforms.

Sialic acid residues are likely essential for apoE-HDL association. A decrease in apoE4 glycosylation and sialylation affects its lipidation [19]. ApoE4’s morphology and reduced sialylation within its lipid domain (residues 244–275) may explain its preference for larger lipidated particles, such as VLDL, as opposed to the smaller HDL typically associated with the other two isoforms [7, 12, 29]. ApoE4’s reduced binding affinity for HDL may alter its half-life in circulation and its receptor affinity [1, 30, 31]. These variations in binding affinity among the apoE isoforms affect their ability to aid in the clearance and degradation of Aβ via receptor-mediated transcytosis and endocytosis. We have previously reported that small, not large, apoE containing HDL-P in CSF correlate with lower measures of cerebral amyloidosis, as assessed by CSF Aβ_42_ levels [8]. Here, we demonstrate that the percentage of secondary glycosylation correlates with concentrations of s-HDL-P in CSF. ApoE-HDL, perhaps in s-HDL-P, acts as an Aβ chaperone and complexes with the lipoprotein related receptor 1 (LRP1), LDLR, and very low-density lipoprotein receptor (VLDLR) to promote endocytosis of soluble Aβ in astrocytes and microglia, unlike poorly lipidated apoE [5]. Of the three apoE isoforms, apoE4 on larger particles binds with the highest affinity to LDLR and LRP1, which competes with Aβ binding [32]. LRP1 also transports Aβ from the interstitial fluid across the blood–brain barrier (BBB) [33, 34], after which megalin (LRP2) clears Aβ at the choroid plexus across the blood-cerebral spinal fluid barrier (BCSFB) [35].

The greater proportion of cognitively normal *APOE* Ɛ4 carriers, particularly the homozygotes, represented in this cohort is both a strength and weakness. Here, we observed greater CSF total and secondary glycosylation patterns in the MCI vs NCI group and lower CSF secondary glycosylation in AD vs MCI group, likely explained by having more *APOE* Ɛ4 homozygotes in the NCI group (Tables 1 and 2). A similar trend of reduced sialylation in AD was observed in another study [36]. While only the non-Ɛ4 carriers in the MCI group showed greater CSF glycosylation, a limitation in this analysis is posed by the presence of only three Ɛ4 allele carriers in the MCI group (Fig. 1S A-C). Therefore, the difference between non-Ɛ4 and Ɛ4 carriers by clinical status cannot be fully discerned; however, it is important to note that among the Ɛ4 carriers, those with the Ɛ3 Ɛ4 genotype exhibited significantly higher CSF glycosylation percentages than those with the Ɛ4 Ɛ4 genotype (Fig. 2S A&B). Although this distribution of *APOE* Ɛ4 homozygotes by clinical status may not represent the general population, the correlation of *APOE* genotype with ApoE glycosylation was observed before the onset of cognitive impairment.

A greater percentage of total and secondary CSF apoE glycosylation was associated with higher levels of CSF Aβ_42_ (Fig. 3), and with levels of s-HDL-P, a marker of apoE lipidation (Fig. 4). ApoE desialylated affected the degree of Aβ_42_ degradation in an apoE isoform-specific pattern (E3 > E4) in microglia (Fig. 5A). Consistent with our results, Sugano M et al. [11] determined that the sialic acid moiety at Thr^194^ and lipidation of apoE were crucial for Aβ binding using SPR experiments. Hashimoto, T. et al. reported that Aβ oligomerization increased in an isoform-dependent manner (E4 > E3 > E2) [37], and another study reaffirmed the importance of apoE sialic acid moieties in attenuating toxic Aβ species, especially in the presence of the apoE2 isoform [38]. Overall, these findings support a role for lower apoE glycosylation on cerebral Aβ accumulation before the onset of dementia, potentially acting as a risk factor for AD dementia in those carrying the Ɛ4 allele.

Heparan sulfate proteoglycans (HSPGs) recognize apoE’s LDLR binding region and affect Aβ cellular uptake [39]. Interestingly, a newly discovered apoE isoform stemming from a unique mutation in the *APOE3* gene and resulting in the replacement of Arg^136^ with Ser^136^, dubbed Christchurch ApoE3 (*APOE3ch*), was shown to have a 98% reduced affinity for HSPGs and 60% reduced affinity for LDLR, indicating that ApoE3ch has a LDLR binding affinity between apoE2 and apoE3 [40]. However, it was shown that Ɛ2 homozygotes with the Paisa mutation are less resistant to cognitive decline than an inheritor of two ApoEch alleles, thus ruling out LDLR binding as an explanation for its neuroprotective effects. This marked HSPGs as the receptors of interest [41]. The presence of negatively charge sialic acids on apoE repels the negatively charge heparin, thus reducing its binding. HSPGs not only promote Aβ aggregation, but also promote glia cell inflammation response and the propagation of toxic tau species [39]. They further verified that apoE3ch produced the lowest levels of Aβ_42_ toxic oligomers and that apoE3ch had the lowest binding affinity to heparin among the other three isoforms (E2 < E3 < E4) [41]. Here, we demonstrated that the removal of sialic acid moieties increased CSF apoE3 binding to heparin (Fig. 5B).

One of the limitations of this study is that the association of apoE glycosylation and CSF Aβ_42_ are correlative and may not imply causation. However, at least two independent groups identified a direct effect of apoE glycosylation on Aβ binding [11] and oligomerization [38] ex vivo, increasing our confidence that apoE glycosylation has direct effects on Aβ metabolism and could be a potential target for treatments. We provide direct evidence that apoE sialylation promotes oligomeric Aβ_42_ degradation in an apoE isoform-specific pattern (E3 > E4) in microglial cells and affects heparin binding. While this is the largest study to date measuring apoE glycosylation in relation to AD biomarkers, the main limitation to this study is the lack of *APOE* Ɛ4 homozygotes in the MCI and AD groups that would allow us to make further inferences on the effect of CSF apoE glycosylation on cognitive status. We also recognize the limitations of estimating cerebral amyloidosis using CSF Aβ_42_. Postmortem examinations and Aβ PET imaging provide more accurate assessment of cerebral amyloidosis, but were not available for this study. We were limited to using one batch of pooled Ɛ3/Ɛ3 CSF to isolate enough apoE on account of its lower abundance in CSF, and desialylation was not performed on Ɛ4 CSF for heparin blinding experiments.

## Conclusion

ApoE isoform-specific glycosylation in CSF appears to play an important role in apoE-HDL formation, receptor binding, and CSF Aβ_42_ levels in an isoform-dependent manner. As presented, apoE4 glycosylation is a possible biomarker of AD and potential therapeutic target for the prevention of ApoE-associated cerebral amyloidosis. Future research needs to address whether restoring apoE4 glycosylation would enhance Aβ clearance, attenuate toxic tau and Aβ_42_ propagation, and reduce the glia inflammatory response in vivo.

## Supplementary Information

**Additional file 1: Figs. 1S-5S**.

## Data Availability

Biomarker and coded data will be shared with other investigators upon request.
