# Supplementary material for: An association of CSF apolipoprotein E glycosylation and amyloid-beta 42 in individuals who carry the APOE4 allele
Source: Alzheimers Res Ther. 2023 May 23;15:96. doi: 10.1186/s13195-023-01239-0 (PMC10204298; doi:10.1186/s13195-023-01239-0)
Supplement: Supplementary file 1 — Additional file 1: Figs. 1S-5S. [file 13195_2023_1239_MOESM1_ESM.pdf]

An Association of CSF Apolipoprotein E glycosylation and amyloid-beta 42 in individuals who carry the APOE4 allele

Supplementary File

Cristiana J. Meuret[1], Yueming Hu[2], Sabrina Smadi[1], Mikaila Ann Bantugan[1], Haotian Xian[1], Ashley E. Martinez[1], Ronald M. Krauss[3], Qiu-Lan Ma[1], Dobrin Nedelkov [2, a, *], Hussein N Yassine [1, a, *]

[1] University of Southern California, Los Angeles, CA

[2] Isoformix Inc., Phoenix, AZ

[3] University of California, San Francisco, CA

[a] These authors share senior authorship with equal contributions

* To whom correspondence should be addressed: 9830 S. 51. St. Suite B-113, Phoenix AZ 85044, Tel. 602.295.4874, Email: dobrin.nedelkov@isoformix.com; or 2250 Alcazar St, Rm 210, Los Angeles, CA, 90033, Email: hyassine@usc.edu.

Supplementary Figures

Fig. 1S. The % glycosylation of apoE, $A\beta_{42}$, Tau, and pTau, as a function of clinical status (NCI, MCI, and AD).

Fig. 2S. The % glycosylation of apoE by genotype.

Fig. 3S. The % glycosylation of apoE is significantly lower in CSF than plasma. Total apoE glycosylation was computed by dividing the peak intensity of the glycosylated forms with the total apoE peak intensity (n = 106). The groups were compared using a linear regression model.

Fig. 4S. Association between small HDL particles and CSF $A\beta_{42}$ levels.

Fig. 5S. Mass spectra resulting from the MSIA analyses of apoE3 isolated from CSF (fully sialylated), and CSF apoE3 isolated after desilalyation with sialidase. The shift in signal from m/z = 35,179 to m/z = 34,603 is indicative of the removal of two sialic acids.

Figure 1S

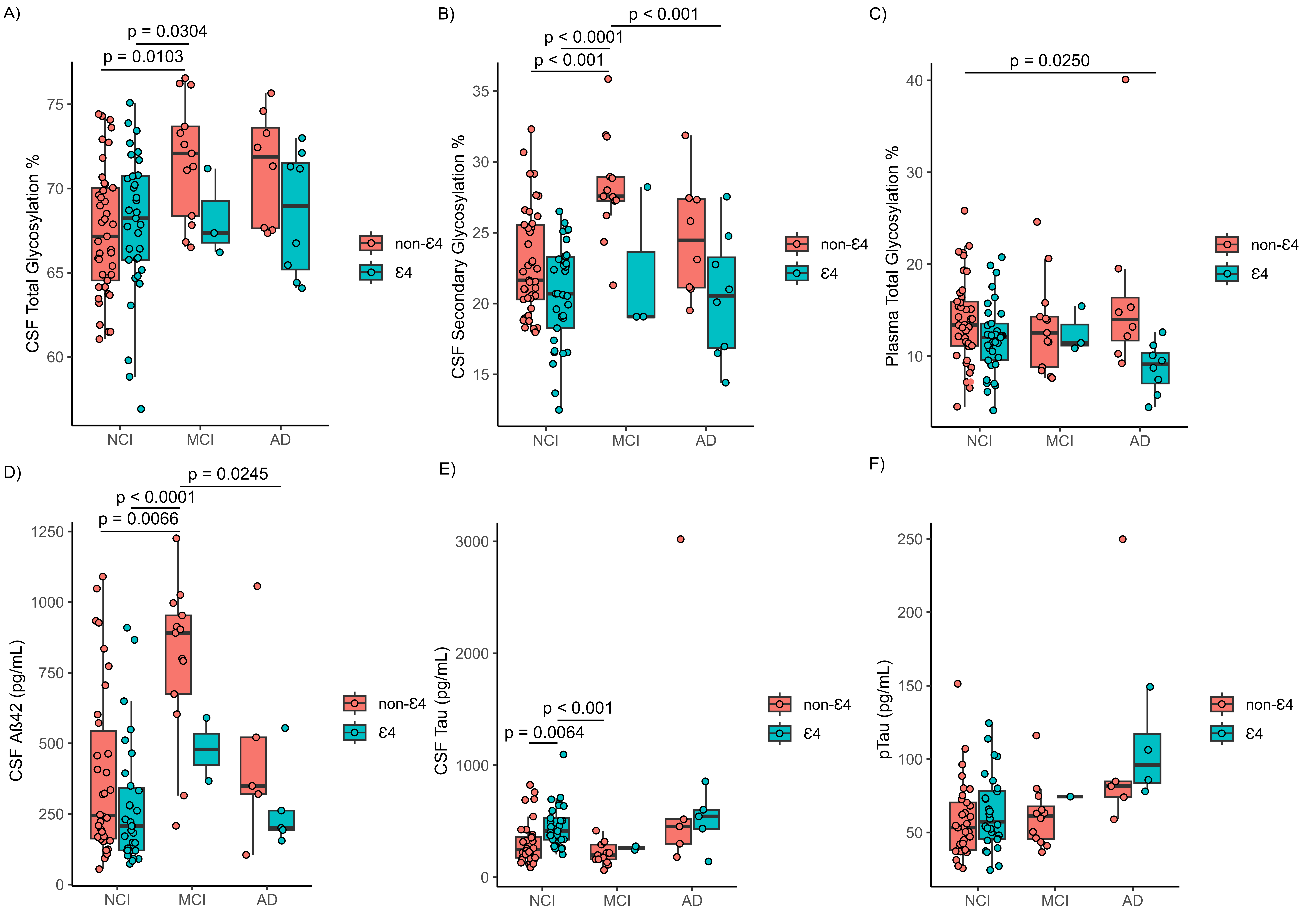
A)
p = 0.0304
p = 0.0103
CSF Total Glycosylation %
75
70
65
60
NCI
MCI
AD
non-Ɛ4
Ɛ4
B)
p < 0.001
p < 0.0001
p < 0.001
CSF Secondary Glycosylation %
35
30
25
20
15
NCI
MCI
AD
non-Ɛ4
Ɛ4
C)
p = 0.0250
Plasma Total Glycosylation %
40
30
20
10
NCI
MCI
AD
non-Ɛ4
Ɛ4
D)
p = 0.0245
p < 0.0001
p = 0.0066
CSF Aß42 (pg/mL)
1250
1000
750
500
250
0
NCI
MCI
AD
non-Ɛ4
Ɛ4
E)
p < 0.001
p = 0.0064
CSF Tau (pg/mL)
3000
2000
1000
0
NCI
MCI
AD
non-Ɛ4
Ɛ4
F)
pTau (pg/mL)
250
200
150
100
50
NCI
MCI
AD
non-Ɛ4
Ɛ4

Figure 2S

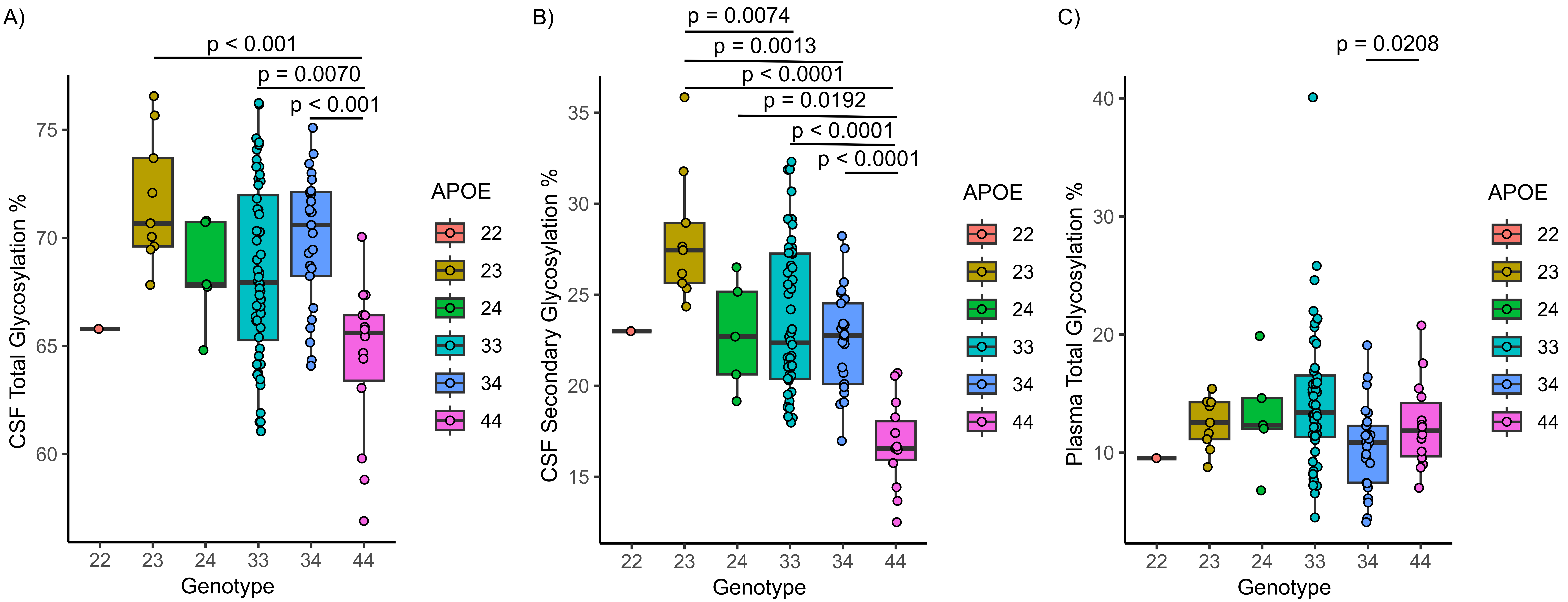

A)
p < 0.001
p = 0.0070
p < 0.001
CSF Total Glycosylation %
75
70
65
60
22
23
24
33
34
44
Genotype
APOE
22
23
24
33
34
44
B)
p = 0.0074
p = 0.0013
p < 0.0001
p = 0.0192
p < 0.0001
p < 0.0001
CSF Secondary Glycosylation %
35
30
25
20
15
22
23
24
33
34
44
Genotype
APOE
22
23
24
33
34
44
C)
p = 0.0208
Plasma Total Glycosylation %
40
30
20
10
22
23
24
33
34
44
Genotype
APOE
22
23
24
33
34
44

Figure 3S

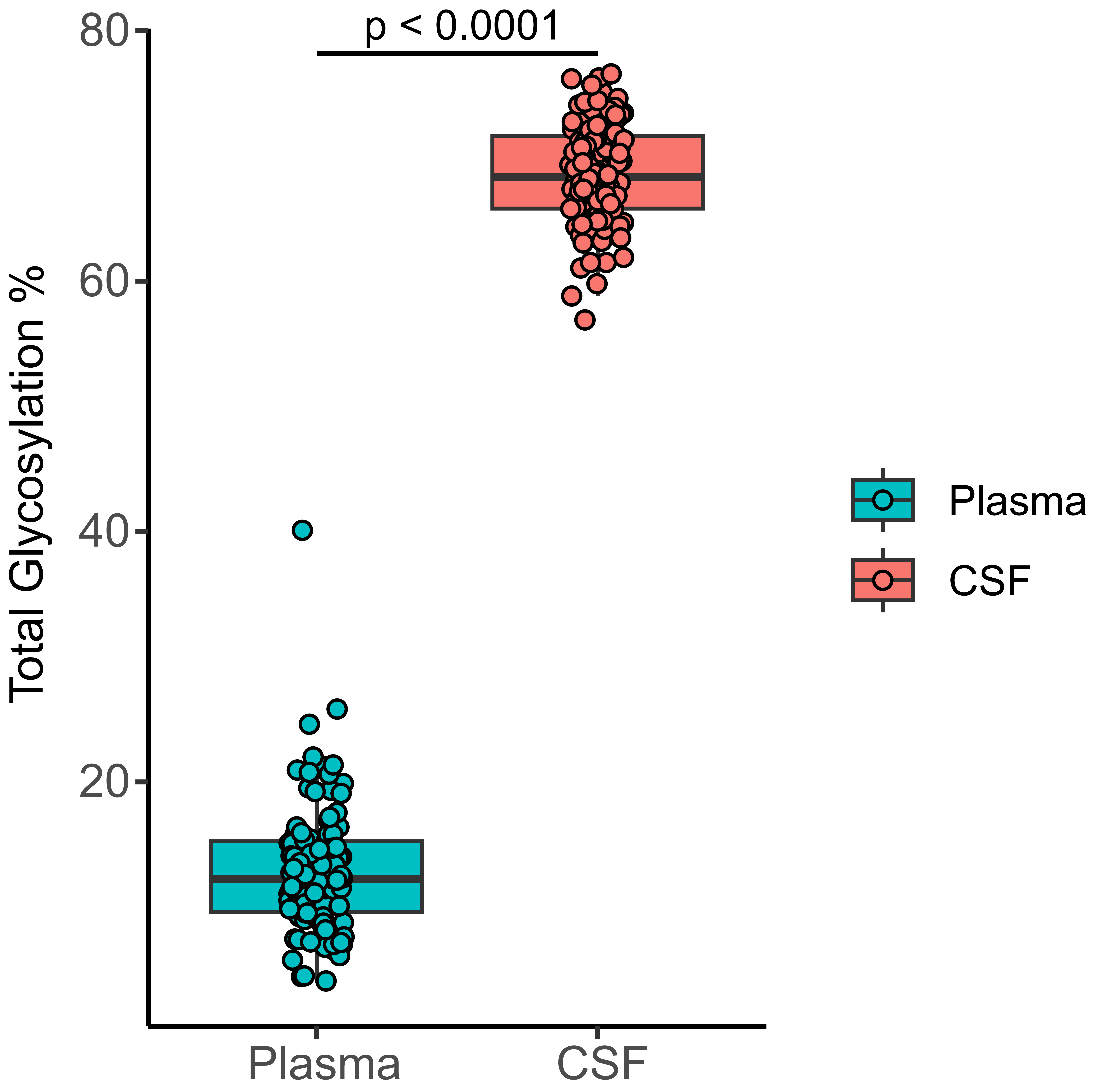
p < 0.0001
80
60
40
20
Total Glycosylation %
Plasma
CSF
Plasma
CSF

Figure 4S

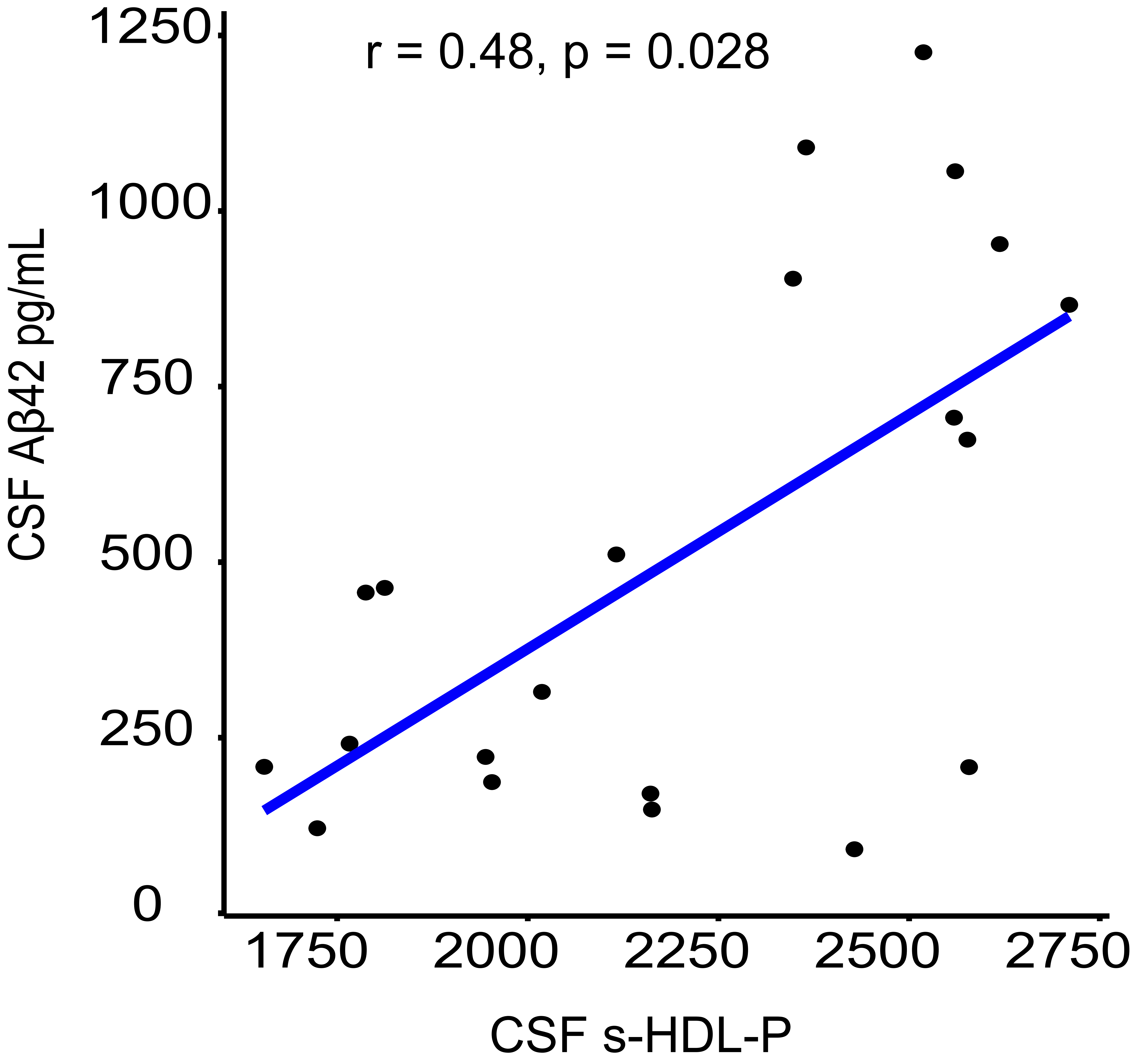

r = 0.48, p = 0.028
1250
1000
750
500
250
0
CSF Aβ42 pg/mL
1750
2000
2250
2500
2750
CSF s-HDL-P

Figure 5S

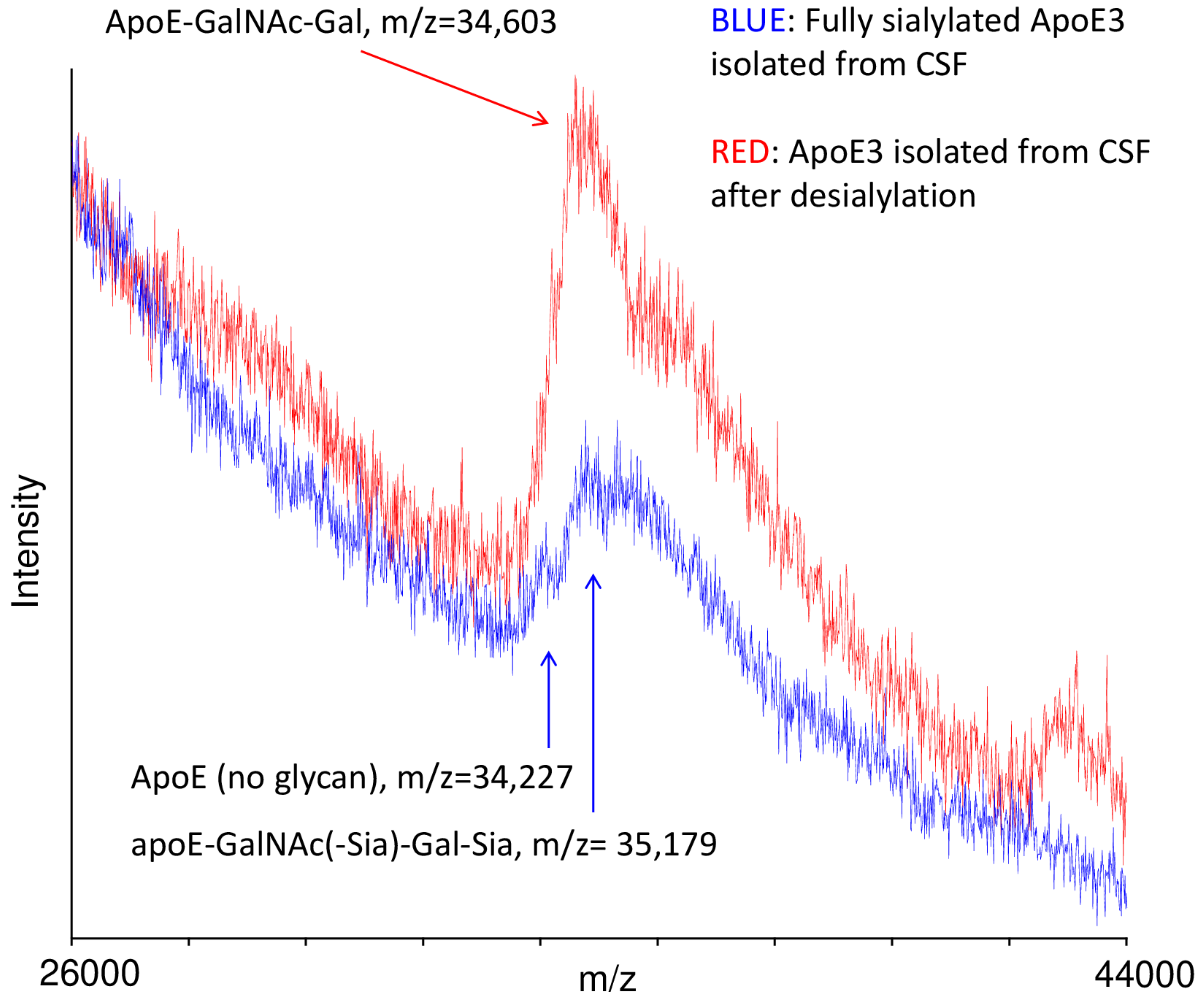

ApoE-GalNAc-Gal, m/z=34,603
BLUE: Fully sialylated ApoE3 isolated from CSF
RED: ApoE3 isolated from CSF after desialylation
Intensity
ApoE (no glycan), m/z=34,227
apoE-GalNAc(-Sia)-Gal-Sia, m/z= 35,179
26000
m/z
44000
